# The fiber diameter traits of Tibetan cashmere goats are governed by the inherent differences in stress, hypoxic, and metabolic adaptations: an integrative study of proteome and transcriptome

**DOI:** 10.1186/s12864-022-08422-x

**Published:** 2022-03-07

**Authors:** Bingru Zhao, Cuiling Wu, Abdul Sammad, Zhen Ma, Langda Suo, Yujiang Wu, Xuefeng Fu

**Affiliations:** 1 National Engineering Laboratory for Animal Breeding, Key Laboratory of Animal Genetics, Breeding, and Reproduction, Ministry of Agriculture, College of Animal Science and Technology, China Agricultural University, Beijing, China; 2 College of Animal Science, Xinjiang Agricultural University, Urumqi, China; 3 Key Laboratory of Genetics Breeding and Reproduction of the Wool Sheep & Cashmere Goat in Xinjiang, Institute of Animal Science, Xinjiang Academy of Animal Sciences, Urumqi, China; 4 Institute of Animal Science, Tibet Academy of Agricultural and Animal Husbandry Sciences, Lhasa, China

**Keywords:** Adaptation, Cashmere, Hypoxia, Proteomic, Tibetan cashmere goat, Transcriptomic

## Abstract

**Background:**

Tibetan cashmere goats are served as a valuable model for high altitude adaptation and hypoxia complications related studies, while the cashmere produced by these goats is an important source of income for the herders. The aim of this study was to investigate the differences in protein abundance underlying the fine (average 12.20 ± 0.03 μm of mean fiber diameter) and coarse cashmere (average 14.67 ± 0.05 μm of mean fiber diameter) producing by Tibetan cashmere goats. We systematically investigated the genetic determinants of fiber diameter by integrated analysis with proteomic and transcriptomic datasets from skin tissues of Tibetan cashmere goats.

**Results:**

We identified 1980 proteins using a label-free proteomics approach. They were annotated to three different databases, while 1730 proteins were mapped to the original protein coding genes (PCGs) of the transcriptomic study. Comparative analyses of cashmere with extremely fine vs. coarse phenotypes yielded 29 differentially expressed proteins (DEPs), for instance, APOH, GANAB, AEBP1, CP, CPB2, GPR142, VTN, IMPA1, CTSZ, GLB1, and HMCN1. Functional enrichment analysis of these DEPs revealed their involvement in oxidation-reduction process, cell redox homeostasis, metabolic, PI3K-Akt, MAPK, and Wnt signaling pathways. Transcription factors enrichment analysis revealed the proteins mainly belong to NF-YB family, HMG family, CSD family. We further validated the protein abundance of four DEPs (GC, VTN, AEBP1, and GPR142) through western blot, and considered they were the most potential candidate genes for cashmere traits in Tibetan cashmere goats.

**Conclusions:**

These analyses indicated that the major biological variations underlying the difference of cashmere fiber diameter in Tibetan cashmere goats were attributed to the inherent adaptations related to metabolic, hypoxic, and stress response differences. This study provided novel insights into the breeding strategies for cashmere traits and enhance the understanding of the biological and genetic mechanisms of cashmere traits in Tibetan cashmere goats.

**Supplementary Information:**

The online version contains supplementary material available at 10.1186/s12864-022-08422-x.

## Background

The Tibetan cashmere goat (*Capra hircus*) is a critical source of cashmere and meat production and significantly contributes to the rural economy. The white cashmere goats of north-western Tibet trace their origins to cashmere goats in Kashmir valley. Cashmere derived from the secondary hair follicles, growing by hair development cycle, is a valuable commodity [1–3]. These goats are well-adapted to harsh climatic and hypoxic conditions of high altitudes owing to their dense coat. Due to these inherent characteristics, cashmere goats and other native species like Tibetan yaks and dogs are considered ideal models to study the genetic mechanisms underlying hypoxia related complications and high altitude adaptation [4–7]. The fiber diameter and production of cashmere determine the economic value. With an increase in demand, breeding cashmere goats for fineness and high quality has become a priority.

Several studies have been carried out on transcriptome and methylome to delineate the genetic basis of hair follicle development in cashmere goats and identified potential candidate genes [1, 8]. For instance, Wang et al. [8] revealed the regulatory mechanisms of hair follicle morphogenesis in Cashmere goats. Zhang et al. [9] investigated the hair follicle cycling in milk and cashmere goats. Meanwhile, our previous study reported potential mRNAs and lncRNAs associated with cashmere fineness [10]. These studies significantly contributed to our understanding of the genetic mechanisms underlying the complex biological landscape of cashmere.

From the biological perspective, transcriptome represents the intermediate state of gene expression and can reflect the mechanism such as transcriptional and post-transcriptional regulation, whereas methylome also plays a role [11]. However, the interpretation of this complex relationship of translation and post-translational modifications and its final outcome in the form of proteins, still remains unexplored. Due to protein being the direct functional executor of the organism, it has irreplaceable advantages through transcriptome and proteome data to obtain the characteristics of differences in gene expression level and protein level of samples.

**Fig. 1:**
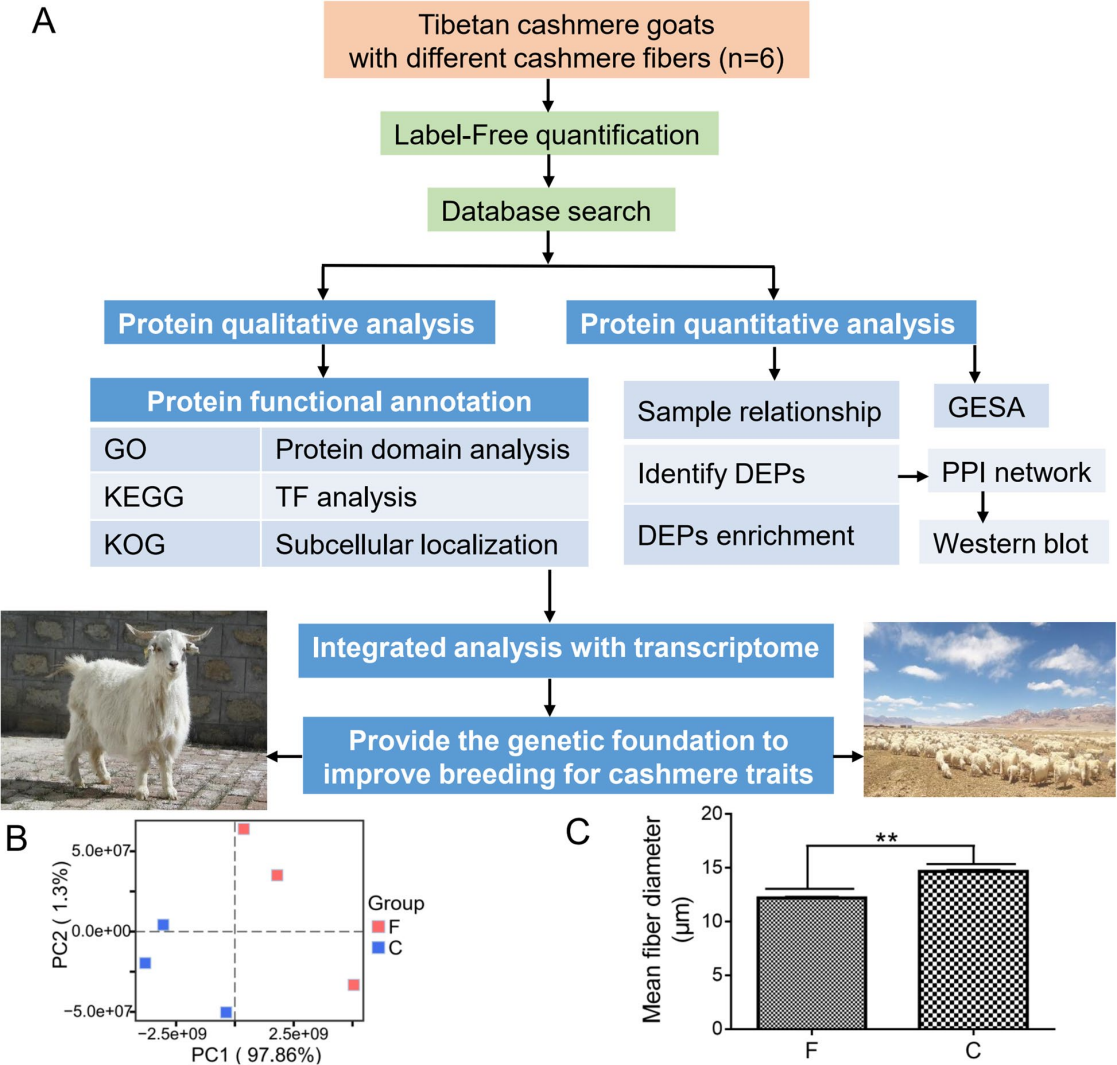
Identification of proteins in the skins of Tibet. **A** The global study design. **B** Mean fibre diameter (MFD) of fineness type (F) cashmere and coarse type (C) cashmere. **C** The principal component analysis (PCA) plot shows separation patterns of skin samples from different mean fibre diameter (MFD) Tibet cashmere goats

Therefore, this study aimed to carry out integrative analysis of proteome with transcriptome (Fig. 1A) to provide novel insights about the final fate of differences observed earlier at the transcriptome level of the fine and coarse cashmere goats. It will be of great value to dissect the critical genes, signaling pathways, and the regulatory mechanism underlying the differences of cashmere fiber diameter in the cashmere goats.

## Results

### Protein identification and functional annotation at different mean fiber diameter (MFD) of cashmere using label-free proteomics

To comprehensively elucidate the proteome profiles that could be involved in the cashmere fiber differences, we identified proteins of skin tissues from Tibetan cashmere goats using label-free quantification proteomics. A total of 12,712 unique peptides were detected (Additional file 1: Tables S1) and 1965 proteins were identified in these skin tissues (Additional file 2: Tables S2). The molecular weight of most the proteins (> 50%) ranged from 0 to 60 kDa (Additional file 3: Fig. S1). In addition, most proteins (> 50%) had high peptide coverage (Additional file 4: Fig. S2). Among the identified proteins, 70% were represented by fewer than five peptides (Additional file 5: Fig. S3), indicating good sequence coverage. Principal component analysis (PCA) demonstrated the difference between the skin tissues of fine-type and coarse-type cashmere goats, indicating our newly generated protein datasets are reliable for further analyses (Fig. 1B, C).

**Fig. 2:**
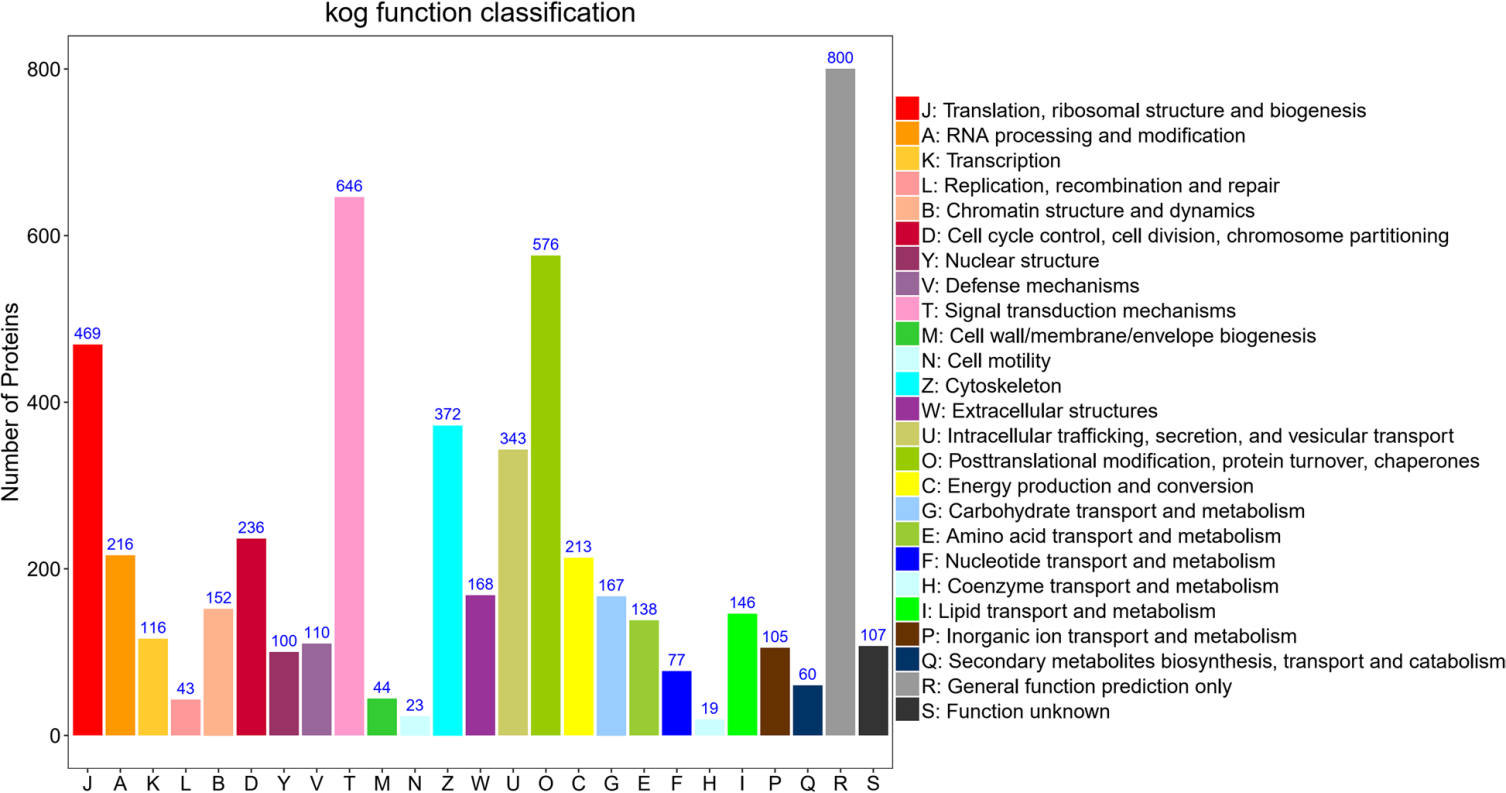
The KOG functional categories of all identified proteins

In order to dissect the function of identified proteins, we further annotated all proteins functions and their classification using three databases. GO annotations revealed that most of the proteins were related to the metabolic and cellular processes (Additional file 6: Fig. S4). Clusters of orthologous groups for eukaryotic complete genomes (KOG) clustering of proteins functional categories included signal transduction mechanisms, posttranslational modification, protein turnover, chaperones, cytoskeleton, cell cycle control, cell division, and chromosome partitioning, indicating these functional categories might be closely related to the regulation of cashmere fiber differences (Fig. 2). Kyoto encyclopedia of genes and genomes (KEGG) pathway analysis (www.kegg.jp/feedback/copyright.html) revealed that most of the proteins participated in one of the carbohydrate metabolism (*n* = 219), signal transduction (*n* = 427), immune system (*n* = 355) (Additional file 7: Fig. S5). A total of 1962 proteins were annotated in these three databases (Additional file 8: Fig. S6).

To search the specific structure and independent functional region of the proteins, we performed protein domain prediction (Additional file 9: Table S3). Overall, the proteins were engaged in the EGF-like domain, laminin EGF domain, immunoglobulin domain, which showed that these candidate genes might play important roles in cashmere traits. To investigate whether the protein is a transcription factor (TFs) and which transcription factor family belongs to, we compared the predicted protein sequence with the corresponding animal TFdb (Additional file 10: Fig. S7). It revealed that the proteins mainly belong to the NF-YB family (*n* = 21, ranked top1), HMG family (*n* = 19, ranked top2), CSD family (*n* = 8, ranked top3). We also investigated the specific location of the proteins in the cell by subcellular localization (Additional file 11: Table S4).

### Identification and functional enrichment of differentially expressed protein (DEPs)

**Fig. 3:**
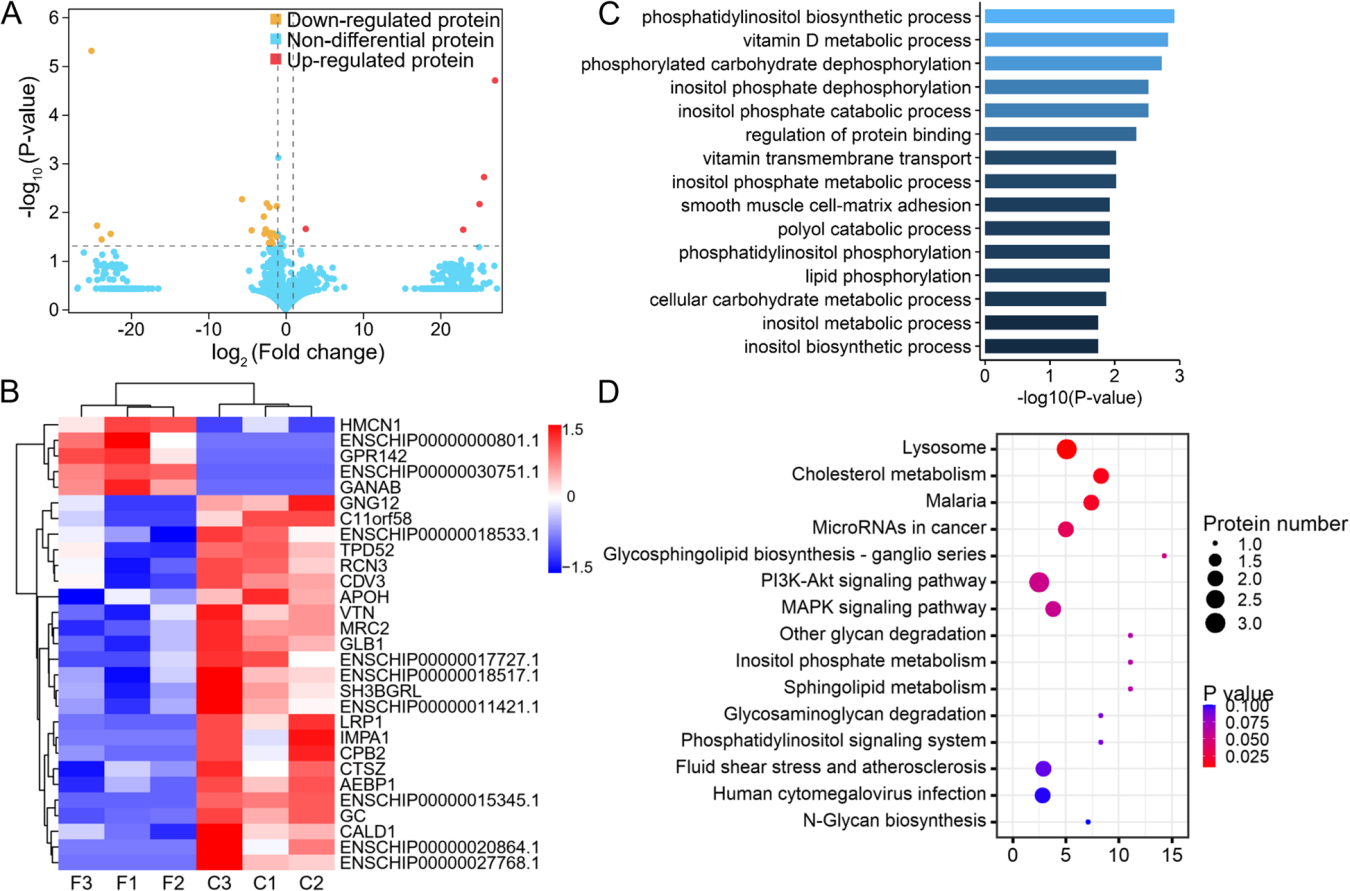
Identification of differential expressed proteins (DEPs) between the fine-type (F) cashmere and coarse-type (C) cashmere skin tissues in Tibetan Cashmere goats. **A** Volcano plot for DEPs. The y-axis corresponds to the mean expression value of log_10_ (*P*-value), and the x-axis displays the log_2_ fold change value. The red dots represent the significantly up-regulated DEPs (fold change > 1.2 and FDR < 0.05), and the yellow dots represent the significantly down-regulated DEPs (fold change < 1.2 and FDR < 0.05); the red dots represent the non-DEPs. **B** Heatmap showed the expression patterns for DEPs. **C** and **D** The top 10 enriched gene ontology (GO) terms (biological processes, BP) and kyoto encyclopedia of genes and genomes (KEGG) pathways on the DEPs

We identified 29 DEPs between the fine-type cashmere and coarse-type cashmere skin tissues in Tibetan cashmere goats, including 5 up-regulated and 24 down-regulated DEPs (Fig. 3A). The heatmap was visualized to investigate the expression profiles patterns of all DEPs, and the results indicated that each group was clustered separately (Fig. 3B). In order to better understand the biological function of these DEPs, GO enrichment and KEGG pathway analysis were performed. We found potentially relevant GO terms such as vitamin transmembrane transport (such as GC), regulation of protein binding (VTN), metabolic process (GLB1), regulation of collagen fibril organization (AEBP1), G-protein coupled receptor signaling pathway (GNG12 and GPR142), these GO terms might directly or indirectly contribute to composition and function of cashmere fiber (Fig. 3C). KEGG pathway analysis (www.kegg.jp/feedback/copyright.html) revealed DEPs were significantly (*P* < 0.05) enriched for the PI3K-Akt signaling pathway, MAPK signaling pathway, and inositol phosphate metabolism (Fig. 3D).

**Fig. 4:**
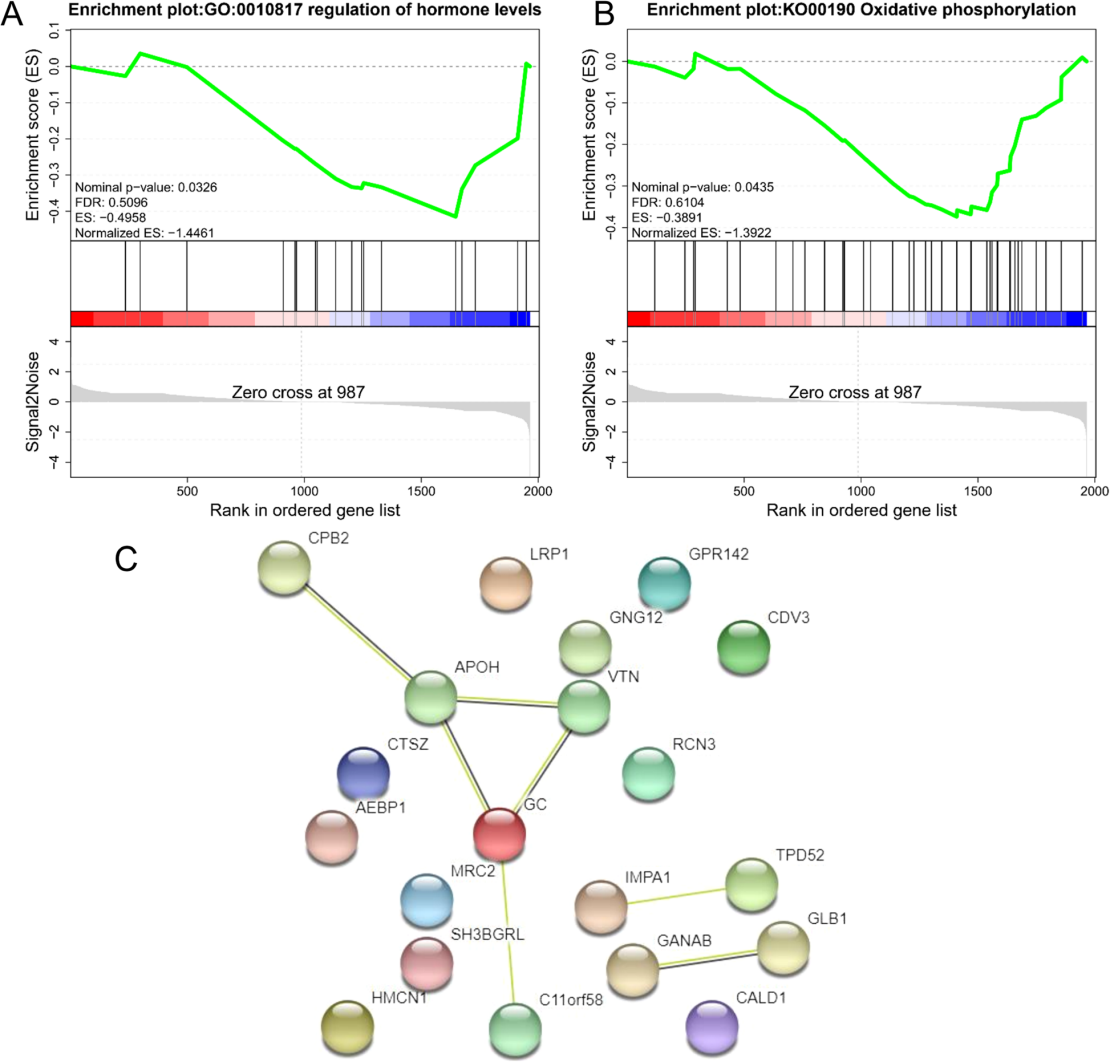
Gene set enrichment analysis (GSEA) of gene ontology (GO) terms (**A**) and kyoto encyclopedia of genes and genomes (KEGG) pathways (**B**) for proteins in skin tissues of Tibetan Cashmere goats. **C** The protein-protein interaction (PPI) network with differentially expressed proteins (DEPs)

Gene set enrichment analysis (GSEA) of all proteins revealed significantly (*P* < 0.05) enriched GO terms, such as regulation of hormone levels (Fig. 4A), and KEGG pathways, such as oxidative phosphorylation (Fig. 4B). Protein-protein interaction (PPI) analysis were performed for the 29 DEPs (7 edges identified; PPI enrichment *P*-value = 0.0002) (Fig. 4C). The PPI network revealed that GC, VTN, APOH, CPB2, and C11orf58 were the core nodes.

### Integrated analysis of proteome and transcriptome

**Fig. 5:**
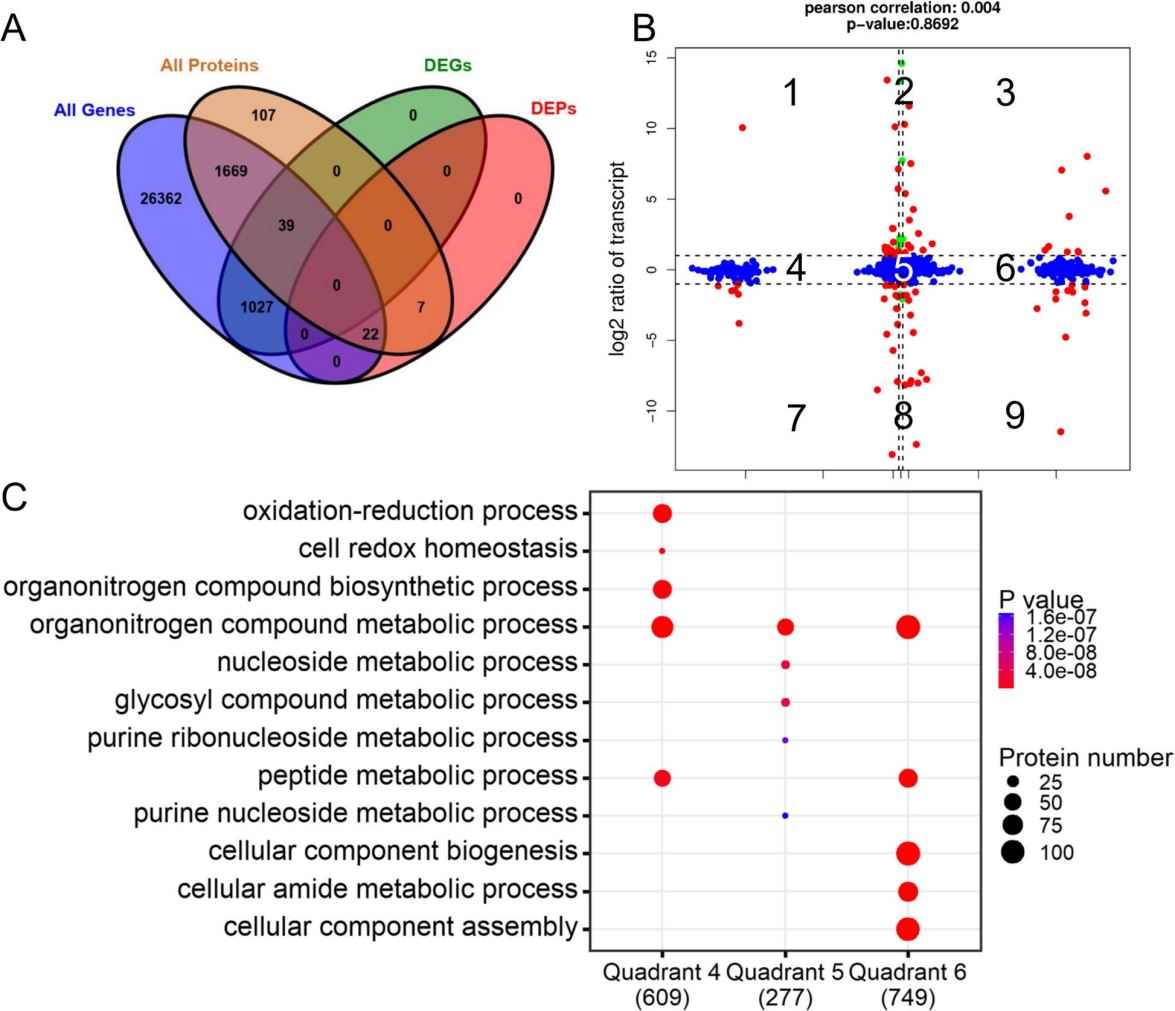
Association analysis of transcriptome and proteome data. **A** Venn plot of the number of all detected genes, all detected proteins, differentially expressed proteins (DEGs), differentially expressed proteins (DEPs). **B** Comparison of the expression between transcriptomic (y-axis) and proteomic (x-axis) profiling. Log_2_ expression ratios were calculated from the expression of fineness type (F) cashmere and coarse type (C) cashmere. Significant changes in expression are color-coded: green, transcripts only; blue, proteins only; red, both. **C** The top 5 enriched gene ontology (GO) terms (biological processes, BP) for genes in quadrants 4, 5, 6 regions

As the transcriptomic data was obtained from the same samples of our previous study [10], we characterized the differences in gene expression and protein levels of these samples. The number of associations observed between transcriptome and proteome is shown in Fig. 5A. The distribution of the corresponding mRNA and proteins were shown through a scatter plot (Fig. 5B). The Pearson correlation coefficient was extremely low (0.004), indicating that the expression levels of mRNA and protein were not always positively correlated in skin tissue of Tibetan cashmere goats. We found a total of 69 concordant dots, representing a corresponding expressed trend between protein abundance and transcript abundance (red dots). In addition, 6 green dots (transcripts only) and 46 blue dots (proteins only) were identified, indicating differential expression was either found on the transcript or the protein levels in our study. We further investigated the functions of proteins in quadrants 4, 5, 6 regions; where most of them are engaged in the oxidation-reduction process, cell redox homeostasis, metabolic and cellular-related processes (Fig. 5C).

### Validation of the expression levels of DEPs

**Fig. 6:**
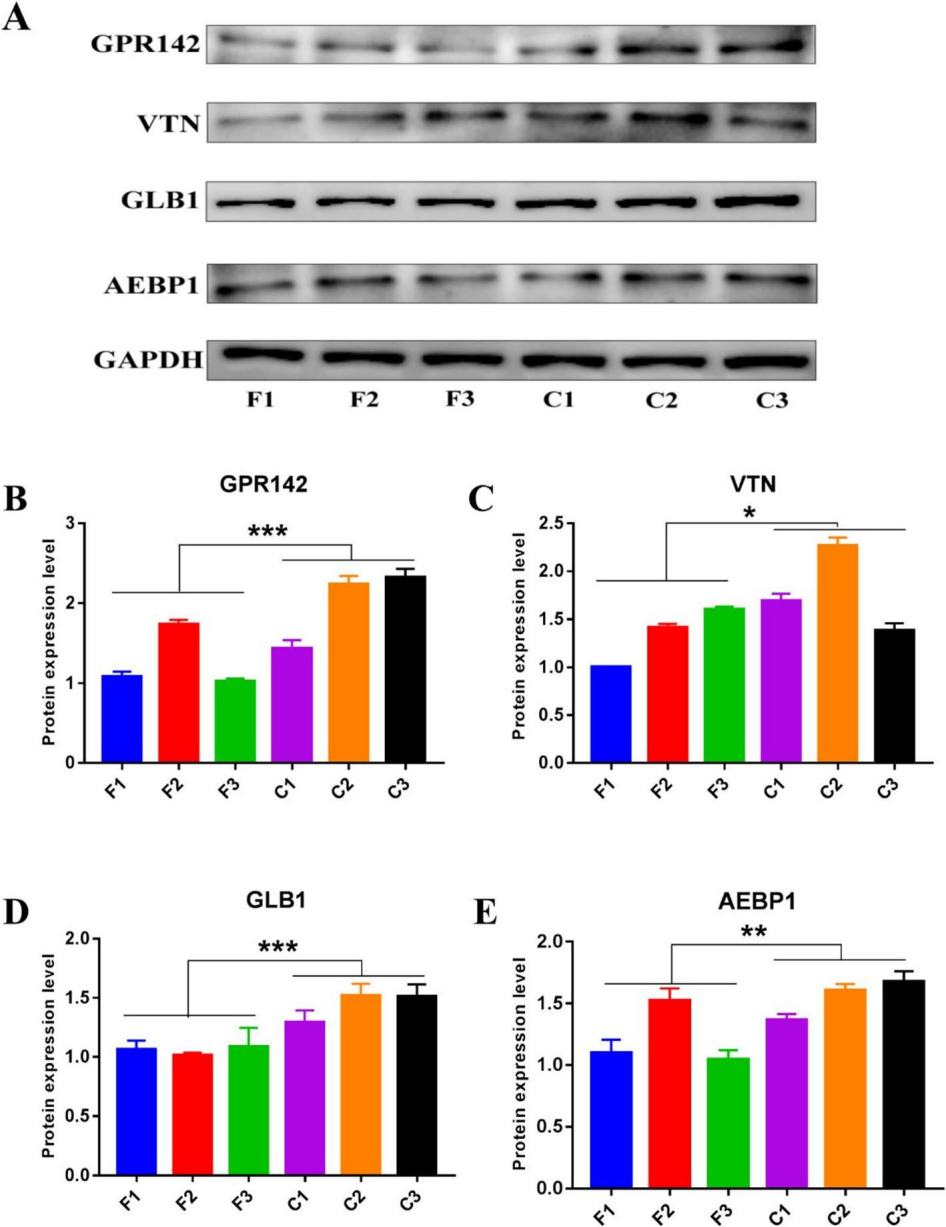
Western blotting and quantitative analysis in the skin tissues of Tibetan cashmere goats. **A** Western blot bands of GPR142, VTN, GLB1, AEBP1, GAPDH was used as control; the experiments were performed using skin tissues for each group and repeated three times. The F1, F2 and F3 correspond to fine type cashmere (F) samples, C1, C2 and C3 correspond to coarse type cashmere (C) samples, respectively. The same as below. **B**–**E** Relative protein expression levels of GPR142, VTN, GLB1 and AEBP1. All data are presented as means ± SE. * represents *P* < 0.05, ** represents *P* < 0.01 and *** represents *P* < 0.001, which regarded as statistically significant, highly significant and extremely significant, respectively

To verify the accuracy of the DEPs detected by proteome, we selected VTN, GLB1, AEBP1, and GPR142 for western blot analyses (Fig. 6A, Additional file 12: Fig. S8). The results showed that the protein abundance changes were consistent with those obtained by proteomics, and the protein expression levels of GPR142, VTN, GLB1, AEBP1 in the C group were significantly higher than F group (*P* < 0.05) (Fig. 6B-E).

## Discussion

Goat cashmere fiber has unique attributes for a valuable model that allow detailed study for hair follicles developmental biology, while the MFD greatly influences the quality of cashmere. In our proteomics study, these 29 DEPs were primarily involved in biological processes related to metabolism, cell process, and environmental information processing. Interestingly, six DEGs members of the solute-carrier family were observed in our previous RNA-seq study [10], and only *SLC2A1* encoding glucose transporter protein type 1 (GLUT1) was upregulated and. Given the importance of GLUT1 in transporting glucose to the cell and vitality for its growth and proliferation [12]; it could be assumed that metabolic level changes might be central to the cashmere phenotype difference in the F and C group. This assumption of metabolic differences between both groups may be further augmented by the fact that hypoxia induces glucose uptake and metabolism through up-regulation of GLUT1 protein [13]. Meanwhile, Tibetan cashmere goats are widely studied for hypoxia and high altitude biology studies [4, 5]. As it is well-known that the upregulation trend of genes is a response to certain external stimulate. Our previous DEGs analysis has not depicted such big difference between both groups [10]. Therefore, it can be assumed from our proteomics approach that the inherent resilience of the F group is pronounced compared to the C group. These changes may be attributed to the role of post-transcriptional, translational, and post-translational modifications and regulatory processes [14], in addition to the regulation of protein degradation by hidden proteases regulatory activity [15]. These phenomena signify the magnitude of variation underlying the phenotype difference between the two groups.

The KEGG enriched proteins majority falls in metabolic, focal adhesion, Wnt signaling pathway, MAPK signaling pathway, inositol phosphate metabolism pathway, and PI3K-Akt signaling pathways. As cashmere goats are used as a model of hypoxia related studies, it is interesting to note a study showing metabolic changes attributed to intrauterine hypoxia impacting the hair metabolome [16]. The focal adhesion pathway is important in cell adherence and its interaction with the extracellular matrix, and thereby has a demonstrated role in hair follicle growth [17, 18]. Wnt pathway involved in hair follicle induction and regulates its downstream morphogenesis and differentiation [19, 20], is upregulated by the hypoxic conditions and hypoxia inducible factor 1-alpha (*HIF-1α*) gene [21–24]. These mechanisms have been shown to regulate metabolic processes contributing to cell survival advantages. PI3K-Akt pathway has also been shown to be crucially involved in hair follicle regeneration, and is regarded as a potential regulating pathway for hair regeneration therapy [25–27]. MAPK pathway essentially regulates the hair cycle and the self-renewal of hair follicle stem cells [28]. Besides, potentially GO terms were identified in this study such as vitamin D metabolic process (GC), regulation of protein binding (VTN), vitamin transmembrane transport (GC), regulation of collagen fibril organization (AEBP1), G-protein coupled receptor signaling pathway (GNG12 and GPR142); these GO terms directly or indirectly regulate and contribute to composition and function of the hair fiber. For instance, vitamin D regulates epidermal differentiation, hair follicle cycling, and hair growth [29]; and G-protein coupled receptor signaling pathway has an important role in hair follicle stem cell activation [30].

Proteins transcription factors prediction also revealed important insights about the unique characteristics of Tibetan cashmere goats. HMG box family of reductase inhibitors actively downregulate the activation of transcription factors NF-kappaB (NF-κB), activating protein-1 (AP-1), and HIF-1α [31, 32]. This phenomenon is noteworthy since NF-κB and HIF-1α are typically involved in metabolic process activation and survival oriented changes during hypoxia [33]. While studies have also shown the downstream regulation of the Wnt signaling pathway through the HMG box family of proteins [34]. NF-YB transcription factor family is widely studied in plants, where it has been related to root growth, drought, and heat resistance [35, 36]; however, related studies about hair growth or hypoxia lacks in mammals. The induction effects of the CSD family on the expression of HIF-1α and cold shock proteins are well-established [37–39], which highlights the importance of hypoxia and harsh climate resistance in Tibetan cashmere goats.

Among the important significant DEPs, VTN is a glycoprotein, and has multifunctional roles in cell adhesion and migration via peri-cellular proteolysis and growth factors signaling. Studies have reported that VTN is a common constituent of the extracellular deposits, therefore it is assumed as a candidate gene and selected for validation [40, 41]. GLB1 is shown to be directly induced by hypoxic conditions and has a particular role in the context of Tibetan cashmere goats, which are also exposed to hypoxic conditions due to their high altitude habitat [5, 42]. Another example is AEBP1, a potential regulator of cholesterol efflux and MAPK signaling, having higher expression in the telogen of hair growth cycle [43–45]. Similarly, GPR142 is a member of the G-protein coupled receptor signaling pathway, has an important role in hair follicle stem cell activation [30]. The results of protein abundance changes in VTN, GLB1, AEBP1 and GPR142 indicated that the expression profiles determined by our current labeling free proteomics method was reliable, and these candidate genes might be contributing to the difference of MFD in Tibetan cashmere goats.

Integrated transcriptome and proteome analyses are advised as an efficient strategy to explore the mechanisms underlying a biological system [46]. However, the pearson correlation coefficient between DEGs and DEPs from our study was low (0.004). There are instances of previous studies reporting a lower correlation between mRNA and protein data [47, 48]. The reason might be that the expression levels of protein coding genes are not an end in themselves but rather it fulfills the protein synthesis need of the biological systems in its near future. Mammalian cells are shown to produce fewer copies of relatively unstable mRNA at a much lower rate than dozens of stable protein copies per mRNA [49, 50]. Therefore, relative abundances of proteins might or might not occur in proportion to their relative mRNA levels [51, 52].

## Conclusions

In this study, we characterized the protein abundance changes of different cashmere fibers in Tibetan cashmere goats. We highlighted the functional and regulatory networks by integrating proteome with transcriptome. Finally, we provided novel insights into the biological mechanisms underlying the adaptation of Tibetan cashmere goats to hypoxic high altitude elevations and the dissection of the molecular mechanisms underlying the different MFD of cashmere. Differences between inherent metabolic adaptation to harsh and hypoxic conditions may be correlated to the observed phenotype differences. Further studies are warranted in this direction to clarify these complex relationships.

## Methods

### Animals and sample collection

Tibetan cashmere goats were obtained from the goat breeding farm located in Tibet (longitude: 91°12′-93°02′ E, latitude: 30°31′-31°55′ N, altitude: about 5000 m), China. The local environmental challenge to animals is the low oxygen availability at high altitudes. The partial pressure of oxygen at 5000 m is only about 50% of the value at sea-level, and the resultant hypoxia imposes severe constraints on aerobic metabolism [5]. We collected cashmere samples of 50 female goats (1.5 years old) in anagen stage from the left mid-side scapular of each animal body, these female goats were artificially inseminated with fresh sperm from a single ram. The cashmere samples were measured MFD values following the standardized methods set by China Fiber Inspection Bureau. In total, 3 female goats with the lowest MFD values (average 12.20 ± 0.03 μm, 1.5 years old) as the fine type (F) group, and another 3 female goats with the highest MFD values (average 14.67 ± 0.05 μm, 1.5 years old) as the coarse type (C) group, were selected and sampled (Fig. 1B). Scapular skin tissues were collected from these 6 Tibetan cashmere goats in vivo and immediately frozen by liquid nitrogen for further analysis.

### Skin tissue protein extraction and digestion

Total proteins were extracted using the cold acetone method [53]. Protein quality was examined with SDS-PAGE. The results of SDS-PAGE showed that the separated bands were clear, abundant and non-degraded, and the bands were consistent among all samples (Additional file 13: Fig. S9). BCA protein assay kit (Pierce, Rockford, IL) was used to determine the protein concentration of the supernatant The protein concentration of all samples is greater than 1 μg/μL and total protein content is greater than 0.2 mg, meet the label-free proteomics-sequencing requirements (Additional file 14: Table S5). Proteins (50 μg) extracted from cells were suspended in 50 μL solution, reduced by adding 1ul of 1 M dithiotreitol at 55 °C for 1 h, alkylated by adding 5 μl of 20 mM iodoacetamide in the dark at 37 °C for 1 h. The samples were precipitated and washed in acetone and re-suspended in 50 mM ammonium bicarbonate. Finally, the proteins were digested with trypsin (Promega, Madison, WI) at a substrate/enzyme ratio of 50:1 (w/w) at 37 °C for 16 h to obtain peptide mixtures.

### Nano-HPLC-MS/MS analysis

The peptides were re-dissolved in 30 μL solvent A (A: 0.1% formic acid in water) and analyzed by online nano-spray LC-MS/MS on an Orbitrap FusionTM LumosTM coupled to EASY-nLC 1200 system (Thermo Fisher Scientific, MA, USA). The peptide sample was loaded onto the analytical column (Acclaim PepMap C18, 75 μm × 25 cm) and separated with a 120-min gradient, from 5 to 35% B (B: 0.1% formic acid in ACN). The column flow rate was maintained at 200 nL/min with a column temperature of 40 °C. The electrospray voltage of 2 kV versus the inlet of the mass spectrometer was used. The mass spectrometer was run under data dependent acquisition mode and automatically switch between MS and MS/MS modes. The parameters was: (1) MS: scan range (m/z) = 350–1200; resolution = 120,000; AGC target = 400,000; maximum injection time = 50 ms; Filter Dynamic Exclusion: exclusion duration = 30s; (2) HCD-MS/MS: resolution = 15,000; AGC target = 50,000; maximum injection time = 35 ms; collision energy = 32.

### Protein identification and quantification

PEAKS Studio Version X (Bioinformatics Solutions Inc., Waterloo, Canada) mass spectrometry was used for analysis. PEAKS DB was used to search the protein database. PEAKS DB were searched with a fragment ion mass tolerance of 0.05 Da and a parent ion tolerance of 10 ppm. Qualitative analysis of proteins is to determine whether a protein is present in a sample and to identify the type of protein. To ensure the reliability of the results, the peptide false discovery rate (FDR) is required to be less than 1% to evaluate the error discovery rate. In addition, a unique peptide refers to a peptide that had been identified and only comes from a single protein sequence or a sequence from the same group, requiring a protein’s unique peptide ≥1. Peptides and proteins that met these requirements were used for subsequent analysis.

### Functional annotation analysis

The protein functions and classification were analyzed based on searches against the following databases: GO, KOG, and KEGG database (www.kegg.jp/feedback/copyright.html) [54]. Significant GO terms and pathways were examined with a *P* value≤0.05. The predicted transcription factor (TF) sequences were compared by hmmscan with the animalTFDB database [55]. The prediction of the protein domain was used the Pfam_scan [56]. The protein sequence was compared with the Pfam database to obtain the relevant annotation information of protein structure. The software WoLFPSort was used to predict the subcellular location of the protein and study the function of the protein [57].

### Differentially expressed proteins and genes

DEPs analysis was performed by R package edgeR between two groups and FDR method was applied for correction [58]. We identified DEPs with a threshold of fold change>1.2 and a FDR < 0.05. Expressed genes were derived from our previous study [10]. We identified mRNA with a fold change> 2 and a FDR < 0.05 in a comparison as significant DEGs.

### Transcriptome and proteome association analysis

Correlation analysis of genes and proteins was performed by R function cor.test. A nine-quadrant map was drawn based on changes in the expression of the gene in the transcriptome and proteome. Quantitative analysis and enrichment analysis was performed in genes of each region of the nine-quadrant map.

### GSEA and PPI analysis

GSEA was performed by R package GSVA [59] and MSigDB [60] to identify whether a set of genes in specific GO terms/pathways show significant differences in the two groups. Enrichment scores and *P*-value were calculated in default parameters mode. PPI network was identified using String [61], which determined genes as nodes and interaction as lines in a network.

### Western blot analysis of DEPs

Protein samples were subjected to SDS-PAGE, transferred onto PVDF membranes, and blocked with 5% skimmed milk (Boster, Wuhan, China) in TBST for 1 h at room temperature. We used different antibodies for different genes, the spacing was smaller, and the exposure intensity was different, so the blots were cut prior to hybridization with antibodies. Primary antibodies and their dilution ratio were followed as, goat anti-rabbit GPR142 antibody (1:500), goat anti-rabbit VTN antibody (1:1000), goat anti-rabbit GLB1 antibody (1:1000), goat anti-rabbit AEBP1 antibody (1:1000), and goat anti-mouse GAPDH antibody (1:5000) (Proteintech, USA). The protein bands were washed and visualized by using an enhanced chemi-luminescence ECL kit (ThermoFisher Scientific). Band intensities were visualized using Image Lab software with a Bio-Rad system (Bio-Rad, Hercules, CA, USA). The target protein contents were normalized to GAPDH level in each lane. The experiment for each gene was performed in triplicates.

### Statistical analysis

The statistical analyses between two groups were analyzed by Student’s t-test. All values are shown as mean ± SE. For all tests, * represents *P* < 0.05, ** represents *P* < 0.01, and *** represents *P* < 0.001; regarded as statistically significant, highly significant, and extremely significant, respectively.

## Supplementary Information

**Additional file 1: Table S1.** Overview of peptides identification information.

**Additional file 2: Table S2.** Overview of proteins identification information.

**Additional file 3: Figure S1.** The number of proteins identified at various molecular weight ranges.

**Additional file 4: Figure S2.** The distribution of protein’s sequence coverage.

**Additional file 5: Figure S3.** The number of peptide fragments for protein identification.

**Additional file 6: Figure S4.** The GO annotation of all expressed proteins.

**Additional file 7: Figure S5.** The KEGG annotation of all expressed proteins.

**Additional file 8: Figure S6.** Venn plot indicated the overlapped annotated proteins of GO, KEGG, and KOG database.

**Additional file 9: Table S3.** Summary results of protein domain prediction.

**Additional file 10: Figure S7.** The top 10 TF families detected in all expressed proteins.

**Additional file 11: Table S4.** The specific location of the proteins in the cell by subcellular localization.

**Additional file 12: Figure S8.** The target bands and western blots of GPR142, VTN, GLB1, AEBP1. GAPDH was used as control.

**Additional file 13: Figure S9.** The results of SDS-PAGE showed that the separated bands were clear, abundant and non-degraded, and the bands were consistent among all samples. The F1, F2 and F3 correspond to fine type cashmere (F) samples, C1, C2 and C3 correspond to coarse type cashmere (C) samples, respectively.

**Additional file 14: Table S5.** The quantitative results of protein samples.

## Data Availability

The mass spectrometry proteomics data have been deposited to the ProteomeXchange Consortium (http://proteomecentral.proteomexchange.org) via the iProX partner repository with the dataset identifier PXD030146. ‍The RNA sequencing datasets used in the current study were obtained from our previous study [10] and has been deposited in National Center for Biotechnology Information Sequence Read Archive (NCBI SRA) database under BioProject No. PRJNA643003.

## Abbreviations

DEPs: Differentially expressed proteins
DEGs: Differentially expressed proteins
KOG: Clusters of orthologous groups for eukaryotic complete genomes
MFD: Mean fiber diameter
FDR: False discovery rate
GO: Gene ontology
KEGG: Kyoto encyclopedia of genes and genomes
TF: Transcription factor
GSEA: Gene set enrichment analysis
PPI: Protein-protein interaction
PCG: Protein-coding gene
PCA: Principal component analysis
F: Fine type
C: Coarse type
