# Supplementary material for: The fiber diameter traits of Tibetan cashmere goats are governed by the inherent differences in stress, hypoxic, and metabolic adaptations: an integrative study of proteome and transcriptome
Source: BMC Genomics. 2022 Mar 7;23:191. doi: 10.1186/s12864-022-08422-x (PMC8903710; doi:10.1186/s12864-022-08422-x)
Supplement: Supplementary file 3 — Additional file 3: Figure S1. The number of proteins identified at various molecular weight ranges. [file 12864_2022_8422_MOESM3_ESM.pdf]

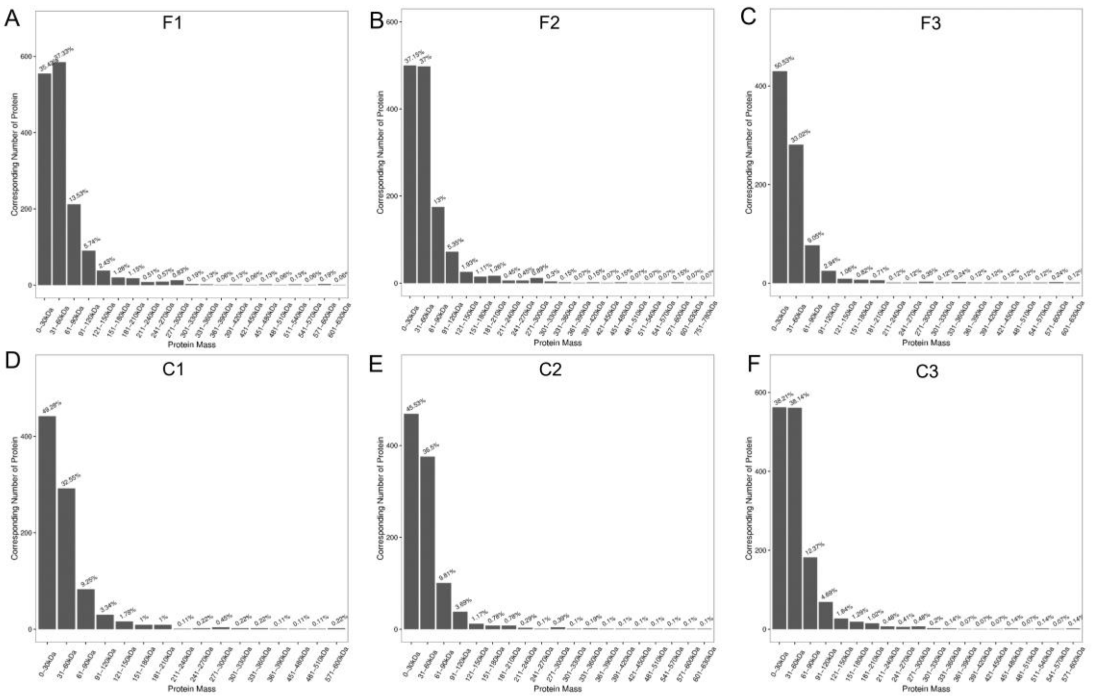


**Additional file 3: Figure S1. The number of proteins identified at various molecular weight ranges.** The x-axis is weight of peptide, and the y-axis is the number of proteins with the number of peptide. The plots A, B, C, D, E, and F indicates F1, F2, F3, C1, C2, and C3 (F, fine; C, coarse) samples, respectively.
