# Supplementary material for: The fiber diameter traits of Tibetan cashmere goats are governed by the inherent differences in stress, hypoxic, and metabolic adaptations: an integrative study of proteome and transcriptome
Source: BMC Genomics. 2022 Mar 7;23:191. doi: 10.1186/s12864-022-08422-x (PMC8903710; doi:10.1186/s12864-022-08422-x)
Supplement: Supplementary file 4 — Additional file 4: Figure S2. The distribution of protein’s sequence coverage. [file 12864_2022_8422_MOESM4_ESM.pdf]

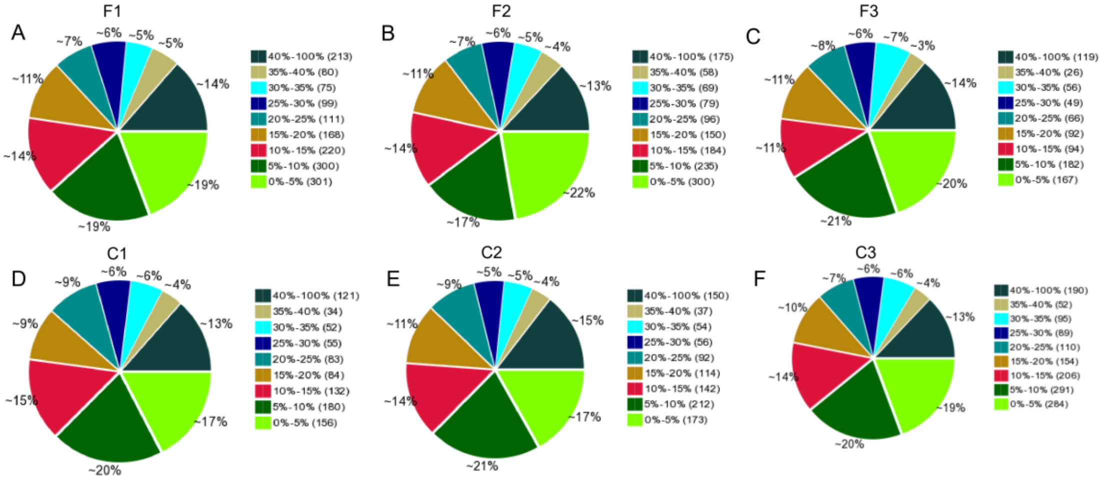

Additional file 4: Figure S2. The distribution of protein's sequence coverage. Different colours represent different ranges of sequences coverage. The percentage shows the proportion of the number of proteins with different coverage ranges/the total number of proteins. The plots A, B, C, D, E, and F corresponds to F1, F2, F3, C1, C2, and C3 (F, fine; C, coarse) samples, respectively.
