# Supplementary figures and images for: The fiber diameter traits of Tibetan cashmere goats are governed by the inherent differences in stress, hypoxic, and metabolic adaptations: an integrative study of proteome and transcriptome
Source: BMC Genomics. 2022 Mar 7;23:191. doi: 10.1186/s12864-022-08422-x (PMC8903710; doi:10.1186/s12864-022-08422-x)

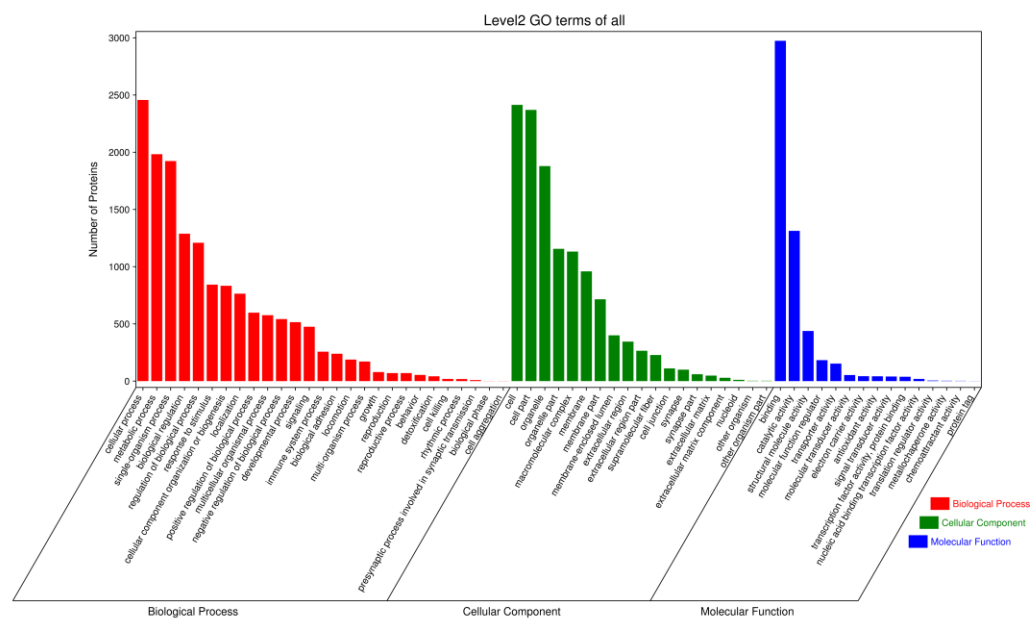

**Additional file 6: Figure S4. The GO annotation of all expressed proteins.**

Supplement: Supplementary file 6 — Additional file 6: Figure S4. The GO annotation of all expressed proteins. [file 12864_2022_8422_MOESM6_ESM.pdf]

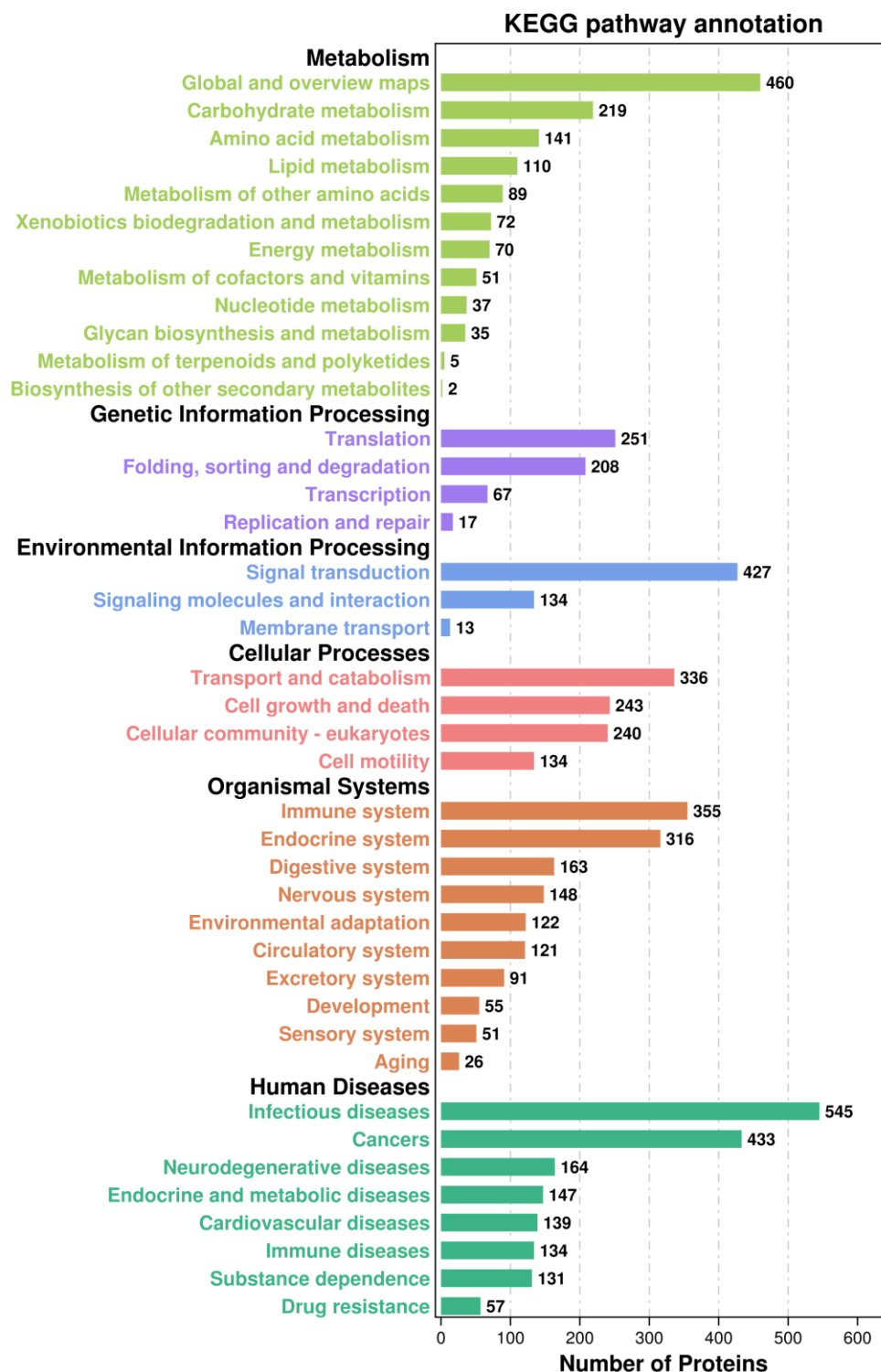

**Additional file 7: Figure S5. The KEGG annotation of all expressed proteins.**

Supplement: Supplementary file 7 — Additional file 7: Figure S5. The KEGG annotation of all expressed proteins. [file 12864_2022_8422_MOESM7_ESM.pdf]

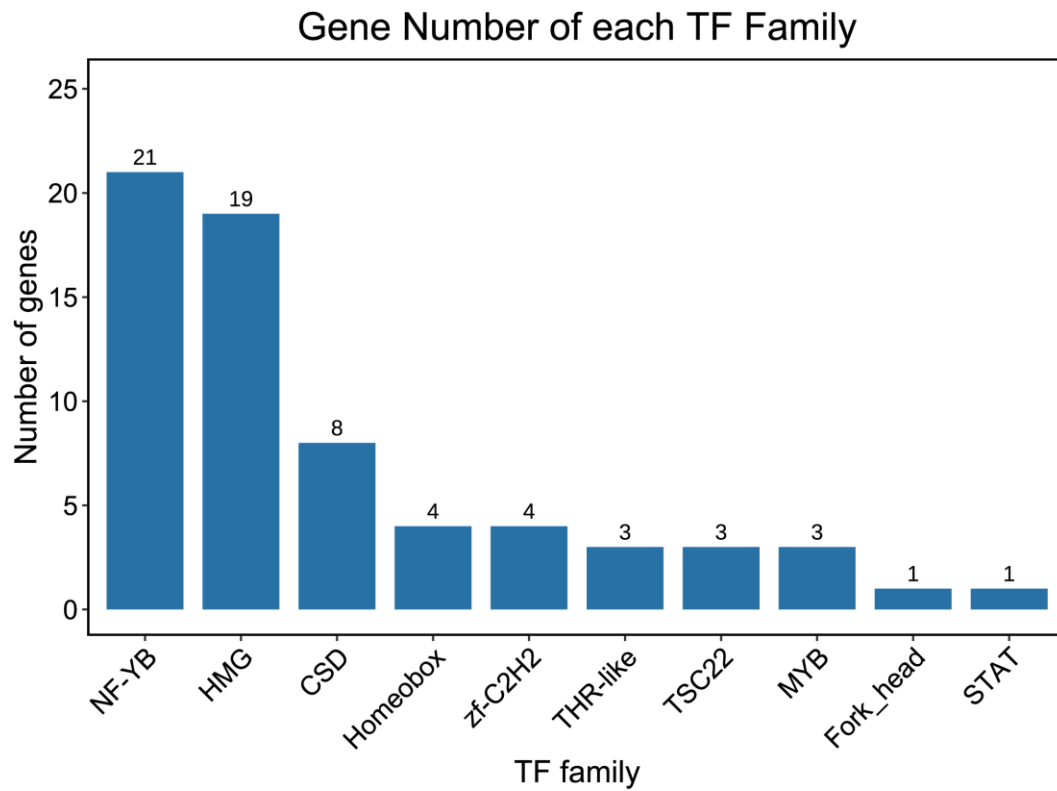

**Additional file 10: Figure S7. The top 10 TF families detected in all expressed proteins.**

Supplement: Supplementary file 10 — Additional file 10: Figure S7. The top 10 TF families detected in all expressed proteins. [file 12864_2022_8422_MOESM10_ESM.pdf]
