# Supplementary material for: The fiber diameter traits of Tibetan cashmere goats are governed by the inherent differences in stress, hypoxic, and metabolic adaptations: an integrative study of proteome and transcriptome
Source: BMC Genomics. 2022 Mar 7;23:191. doi: 10.1186/s12864-022-08422-x (PMC8903710; doi:10.1186/s12864-022-08422-x)
Supplement: Supplementary file 8 — Additional file 8: Figure S6. Venn plot indicated the overlapped annotated proteins of GO, KEGG, and KOG database. [file 12864_2022_8422_MOESM8_ESM.pdf]

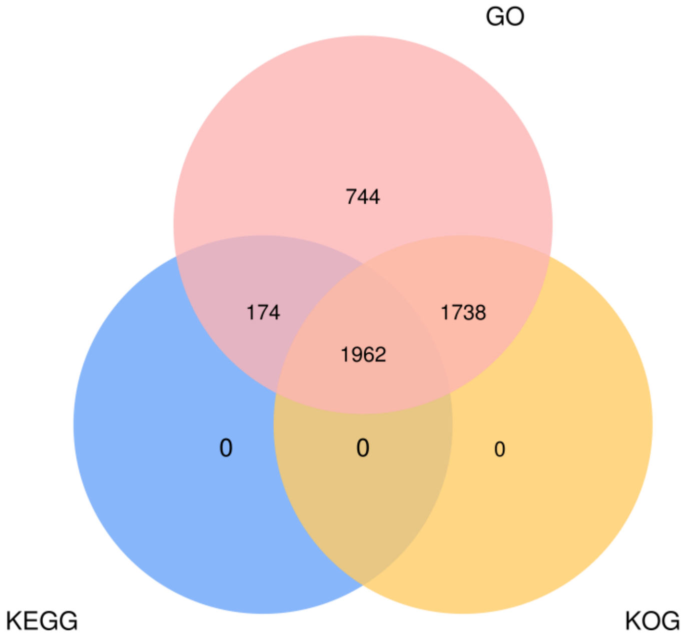

Additional file 8: Figure S6. Venn plot indicated the overlapped annotated proteins of GO, KEGG, and KOG database.
