# Supplementary material for: The fiber diameter traits of Tibetan cashmere goats are governed by the inherent differences in stress, hypoxic, and metabolic adaptations: an integrative study of proteome and transcriptome
Source: BMC Genomics. 2022 Mar 7;23:191. doi: 10.1186/s12864-022-08422-x (PMC8903710; doi:10.1186/s12864-022-08422-x)
Supplement: Supplementary file 12 — Additional file 12: Figure S8. The target bands and western blots of GPR142, VTN, GLB1, AEBP1. GAPDH was used as control. [file 12864_2022_8422_MOESM12_ESM.pdf]

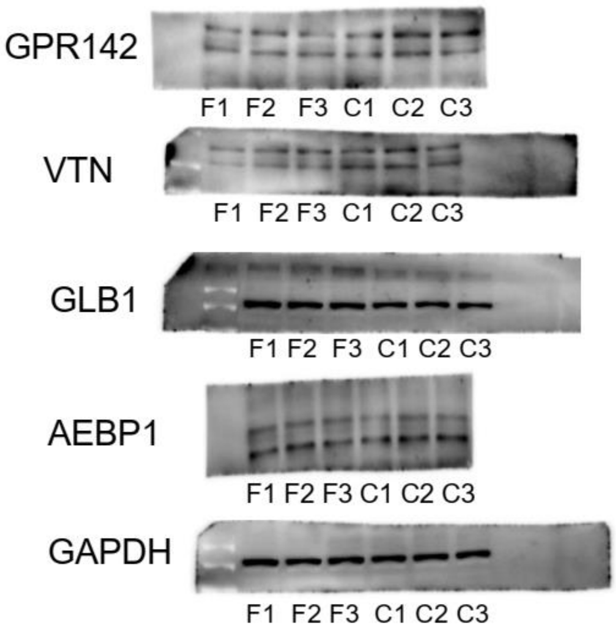

Additional file 12: Figure S8. The target bands and western blots of GPR142, VTN, GLB1, AEBP1. GAPDH was used as control. We used different antibodies for different genes, the spacing was smaller, and the exposure intensity was different, so the blots were cut prior to hybridisation with antibodies. The experiments were performed using skin tissues for each group and repeated three times. The F1, F2 and F3 correspond to fine type cashmere (F) samples, C1, C2 and C3 correspond to coarse type cashmere (C) samples, respectively.
