# Supplementary material for: The fiber diameter traits of Tibetan cashmere goats are governed by the inherent differences in stress, hypoxic, and metabolic adaptations: an integrative study of proteome and transcriptome
Source: BMC Genomics. 2022 Mar 7;23:191. doi: 10.1186/s12864-022-08422-x (PMC8903710; doi:10.1186/s12864-022-08422-x)
Supplement: Supplementary file 13 — Additional file 13: Figure S9. The results of SDS-PAGE showed that the separated bands were clear, abundant and non-degraded, and the bands were consistent among all samples. The F1, F2 and F3 correspond to fine type cashmere (F) samples, C1, C2 and C3 correspond to coarse type cashmere (C) samples, respectively. [file 12864_2022_8422_MOESM13_ESM.pdf]

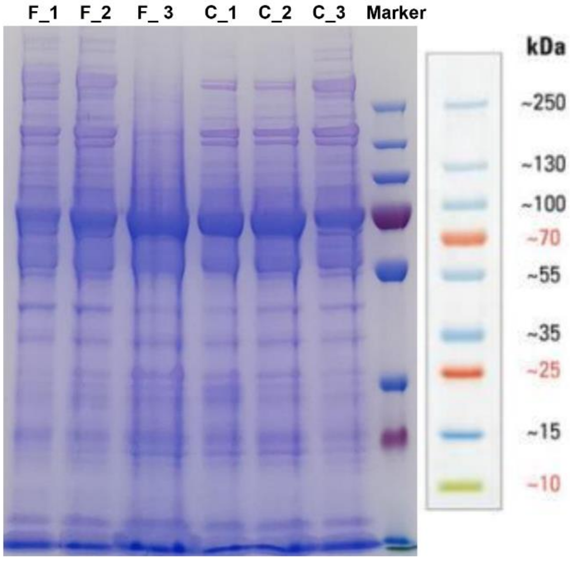


**Additional file 13: Figure S9.** The results of SDS-PAGE showed that the separated bands were clear, abundant and non-degraded, and the bands were consistent among all samples. The F1, F2 and F3 correspond to fine type cashmere (F) samples, C1, C2 and C3 correspond to coarse type cashmere (C) samples, respectively.
